# Association of dietary tomato intake with bladder cancer risk in a prospective cohort of 101,683 individuals with 12.5 years of follow-up

**DOI:** 10.18632/aging.203252

**Published:** 2021-07-09

**Authors:** Xin Xu, Bo Xie, Shiqi Li, Shuo Wang, Dan Xia, Hongzhou Meng

**Affiliations:** 1 Department of Urology, The First Affiliated Hospital, Zhejiang University School of Medicine, Hangzhou 310003, Zhejiang, China

**Keywords:** tomato, lycopene, bladder cancer, cohort, PLCO

## Abstract

Previous studies have provided limited evidence for the effect of tomato intake on bladder cancer incidence. This study aimed to evaluate the association between dietary tomato or lycopene consumption and bladder cancer risk in the Prostate, Lung, Colorectal, and Ovarian Cancer (PLCO) Screening study. Hazard ratios (HRs) and 95% confidence intervals (CIs) were estimated using Cox regression model adjusting for confounders. After a median of 12.5 years of follow-up, 774 incident bladder cancer cases were identified. We found no statistically significant association between dietary intake of raw tomatoes and bladder cancer risk (Adjusted model: HR*_Q5 VS Q1_* = 1.20, 95% CI: 0.95-1.52; *P* for trend = 0.243). Dietary intakes of tomato catsup, tomato salsa and tomato juice were also not associated with the risk of bladder cancer (all *P* for trend > 0.05). There was no statistically significant association between dietary consumption of lycopene and bladder cancer risk (Adjusted model: HR*_Q5 vs. Q1_* = 1.04, 95% CI 0.82-1.33; *P* for trend = 0.590). In summary, analysis of the PLCO study suggested that dietary consumption of tomato or lycopene was not associated with the risk of bladder cancer.

## INTRODUCTION

Bladder cancer is one of the ten most common cancers worldwide with nearly 430,000 cancer cases being diagnosed per year [1]. Despite advances in treatment (e.g., immune checkpoint inhibitor therapeutics), the prognosis remains poor for bladder cancer, especially muscle-invasive tumors [2]. Smoking is the most well-established risk factor with approximately 50% attributable risk of bladder cancer [3]. A small proportion (5-6%) of bladder cancers arise from occupational exposure [4]. Less-established risk factors for bladder cancers include diabetes [5], lack of physical activity [6], obesity [7], nulliparity [8] and high consumption of processed red meat [9].

The potential relationship between dietary tomato/lycopene intake or serum lycopene and bladder cancer risk has been evaluated by a few observational studies and meta-analyses [10–14] with relatively small sample size and inconsistent results. For example, Huang et al. [13] reported that plasma lycopene was significantly inversely associated with bladder cancer risk based on a case-control study from Memorial Sloan-Kettering Cancer Center. By contrast, a recent large meta-analysis failed to find a significant relationship between dietary intake of lycopene or serum lycopene and the incidence of bladder cancer [14]. To contribute to the limited evidence base, we investigated the association between intakes of tomato products or lycopene and the incidence of bladder cancer in the Prostate, Lung, Colorectal, and Ovarian Cancer (PLCO) study.

## RESULTS

There were 774 incident bladder cancer cases after a median follow-up of 12.5 years. Compared to participants who had the largest consumption of raw tomatoes (i.e., quintile 5), participants with the smallest raw tomato intake (i.e., quintile 1), had lower body mass index (BMI), consumed less total energy, and were more likely to be male, Black non-Hispanic or Hispanic, not married, and current smokers, and tended to have an education level of below-college (Table 1).

**Table 1:** Main characteristic of participants in the PLCO cancer screening trial by raw tomato intake.

**Variables**	**Q1 (n=19831)**	**Q2 (n=20560)**	**Q3 (n=20906)**	**Q4 (n=20904)**	**Q5 (n=19482)**	p-value*
Age (y), mean ± SD	62.2 ± 5.4	62.4 ± 5.3	62.5 ± 5.3	62.6 ± 5.3	62.4 ± 5.2	<0.001
Female (n, %)	8924 (45.0%)	10227 (49.7%)	11186 (53.5%)	12215 (58.4%)	9680 (49.7%)	<0.001
Smoking (n, %)						<0.001
Never	9078 (45.8%)	9883 (48.1%)	10233 (48.9%)	10278 (49.2%)	9072 (46.6%)	<0.001
Current	2294 (11.6%)	2000 (9.7%)	1736 (8.3%)	1710 (8.2%)	1650 (8.5%)	
Former	8458 (42.7%)	8674 (42.2%)	8931 (42.7%)	8916 (42.7%)	8757 (44.9%)	
Missing	1 (0.0%)	3 (0.0%)	6 (0.0%)	0 (0.0%)	3 (0.0%)	
Education (n, %)						<0.001
≤High school	9013 (45.4%)	8891 (43.2%)	8455 (40.4%)	8646 (41.4%)	7902 (40.6%)	
≥Some college	10778 (54.3%)	11618 (56.5%)	12410 (59.4%)	12224 (58.5%)	11550 (59.3%)	
Missing	40 (0.2%)	51 (0.2%)	41 (0.2%)	34 (0.2%)	30 (0.2%)	
BMI (n, %)						<0.001
<25.0 kg/m^2^	6599 (33.3%)	6868 (33.4%)	7049 (33.7%)	7099 (34.0%)	6117 (31.4%)	
≥25.0 kg/m^2^	12946 (65.3%)	13409 (65.2%)	13616 (65.1%)	13545 (64.8%)	13101 (67.2%)	
Missing	286 (1.4%)	283 (1.4%)	241 (1.2%)	260 (1.2%)	264 (1.4%)	
Race (n, %)						<0.001
White, Non-Hispanic	16977 (85.6%)	18600 (90.5%)	19402 (92.8%)	19454 (93.1%)	18036 (92.6%)	
Other	2844 (14.3%)	1953 (9.5%)	1500 (7.2%)	1443 (6.9%)	1437 (7.4%)	
Missing	10 (0.1%)	7 (0.0%)	4 (0.0%)	7 (0.0%)	9 (0.0%)	
Drinking (n, %)						<0.001
Never	1928 (9.7%)	2039 (9.9%)	2024 (9.7%)	2141 (10.2%)	1981 (10.2%)	
Former	3380 (17.0%)	3066 (14.9%)	2750 (13.2%)	2872 (13.7%)	2678 (13.7%)	
Current	13882 (70.0%)	14888 (72.4%)	15601 (74.6%)	15289 (73.1%)	14285 (73.3%)	
Missing	641 (3.2%)	567 (2.8%)	531 (2.5%)	602 (2.9%)	538 (2.8%)	
Total energy intake (kcal/d), mean ± SD	1551.2 ± 712.3	1632.8 ± 688.4	1716.1 ± 685.9	1785.1 ± 707.6	2015.2 ± 802.5	<0.001
Marital status (n, %)						<0.001
Married	14522 (73.2%)	15972 (77.7%)	16738 (80.1%)	16831 (80.5%)	15524 (79.7%)	
Not married	5272 (26.6%)	4539 (22.1%)	4128 (19.7%)	4040 (19.3%)	3931 (20.2%)	
Missing	37 (0.2%)	49 (0.2%)	40 (0.2%)	33 (0.2%)	27 (0.1%)	

PLCO, Prostate, Lung, Colorectal, and Ovarian Cancer; Q, quintile; y, year; SD, Standard deviation; BMI, body mass index.

*The p-values were calculated from the comparison of all the 5 groups.

In the multivariate analysis model, there was no statistically significant association between consumption of raw tomatoes and bladder cancer incidence (Table 2, HR*_Q5 VS Q1_* = 1.20, 95% CI: 0.95-1.52; *P* for trend = 0.243). The corresponding adjusted HR was 1.06 (95% CI 0.99-1.13) per 1 SD increment of raw tomato intake. Likewise, dietary intakes of tomato catsup, tomato salsa and tomato juice were not associated with the risk of bladder cancer in the multivariate analysis (all *P* for trend > 0.05). This is also true for the association between dietary consumption of lycopene and bladder cancer risk (Adjusted model: HR*_Q5 vs. Q1_* = 1.04, 95% CI 0.82-1.33; *P* for trend = 0.590). These associations were not modified by potential confounders, including age, sex, race, education level, drinking habits, smoking status and BMI (all *P* for interaction > 0.05).

**Table 2:** Association between tomato/lycopene intake and bladder cancer risk in the PLCO cancer screening trial.

**Variables**	**Median (g/day)**	**Cohort (n)**	**Cases (n)**	**Crude HR (95% CI), p-value**	Adjusted HR (95% CI)*, p-value
Raw tomato (g/day)					
Q1 (≤ 3.57)	1.63	19831	143	Reference	Reference
Q2 (≥ 3.60 to ≤ 9.41)	6.33	20560	165	1.10 (0.88-1.38), p=0.401	1.17 (0.93-1.46), p=0.176
Q3 (≥ 9.44 to ≤ 17.56)	12.91	20906	156	1.02 (0.81-1.28), p=0.880	1.14 (0.91-1.43), p=0.267
Q4 (≥ 17.67 to ≤ 32.44)	23.79	20904	153	1.00 (0.79-1.25), p=0.973	1.19 (0.94-1.50), p=0.151
Q5 (≥ 32.64)	50.24	19482	157	1.11 (0.88-1.39), p=0.381	1.20 (0.95-1.52), p=0.118
				p for trend = 0.571	p for trend = 0.243
Per 1 SD increment				1.02 (0.95-1.09), p=0.551	1.06 (0.99-1.13), p=0.122
Tomato catsup (g/day)					
Q1 (≤ 0.11)	0	21591	154	Reference	Reference
Q2 (≥ 0.13 to ≤ 0.44)	0.17	19540	110	0.79 (0.62-1.01), p=0.058	0.90 (0.70-1.15), p=0.383
Q3 (≥ 0.48 to ≤ 1.15)	0.58	21219	132	0.87 (0.69-1.10), p=0.240	0.91 (0.72-1.15), p=0.443
Q4 (≥ 1.20 to ≤ 2.53)	1.99	19460	198	1.42 (1.15-1.75), p=0.001	0.95 (0.76-1.18), p=0.629
Q5 (≥ 2.95)	5.06	19873	180	1.28 (1.03-1.58), p=0.026	0.96 (0.77-1.20), p=0.712
				p for trend < 0.001	p for trend = 0.887
Per 1 SD increment				1.09 (1.05-1.14), p<0.001	1.05 (0.99-1.11), p=0.088
Tomato salsa (g/day)					
Q1 (= 0)	0	26673	231	Reference	Reference
Q2 (≥ 0.10 to ≤ 0.38)	0.38	19279	114	0.68 (0.54-0.85), p=0.001	1.20 (0.95-1.51), p=0.129
Q3 (≥ 0.39 to ≤ 1.13)	1.10	18951	164	0.98 (0.80-1.20), p=0.853	0.87 (0.71-1.06), p=0.172
Q4 (≥ 1.23 to ≤ 2.99)	1.43	19494	148	0.87 (0.71-1.07), p=0.179	1.06 (0.85-1.31), p=0.616
Q5 (≥ 3.52)	9.33	17286	117	0.78 (0.62-0.97), p=0.029	0.96 (0.76-1.21), p=0.713
				p for trend = 0.181	p for trend = 0.593
Per 1 SD increment				0.98 (0.91-1.06), p=0.612	1.01 (0.94-1.08), p=0.824
Tomato juice (g/day)					
Q1 (= 0)	0	28524	205	Reference	Reference
Q2 (≥ 5.07 to ≤ 5.15)	5.07	17642	122	0.95 (0.76-1.19), p=0.644	0.97 (0.77-1.21), p=0.784
Q3 (≥ 8.12 to ≤ 8.28)	8.28	23990	189	1.08 (0.89-1.32), p=0.434	1.07 (0.87-1.30), p=0.520
Q4 (≥ 11.36 to ≤ 18.19)	13.38	12698	123	1.33 (1.06-1.66), p=0.013	0.94 (0.75-1.18), p=0.596
Q5 (≥ 18.54)	52.27	18829	135	1.00 (0.80-1.24), p=0.968	1.04 (0.83-1.29), p=0.759
				p for trend = 0.891	p for trend = 0.773
Per 1 SD increment				1.03 (0.97-1.09), p=0.376	0.99 (0.92-1.07), p=0.818
Lycopene (mcg/day)					
Q1 (≤ 2791.29)	2074.73	20337	147	Reference	Reference
Q2 (≥ 2791.35 to ≤ 4059.34)	3416.12	20337	157	1.05 (0.84-1.32), p=0.660	1.02 (0.81-1.28), p=0.869
Q3 (≥ 4059.38 to ≤ 5611.78)	4756.86	20336	137	0.92 (0.73-1.16), p=0.475	0.88 (0.70-1.12), p=0.309
Q4 (≥ 5611.82 to ≤ 8436.94)	6736.32	20337	153	1.03 (0.82-1.29), p=0.810	0.95 (0.75-1.21), p=0.671
Q5 (≥ 8437.60)	12062.24	20336	180	1.22 (0.98-1.52), p=0.070	1.04 (0.82-1.33), p=0.748
				p for trend = 0.035	p for trend = 0.590
Per 1 SD increment				1.06 (1.00-1.11), p=0.036	1.02 (0.96-1.09), p=0.469

PLCO, Prostate, Lung, Colorectal, and Ovarian Cancer; HR, hazard ratio; CI, confidence interval; Q, quintile; SD, Standard deviation.

*Adjusted for age, sex, race, body mass index, education, smoking status, drinking status, total energy intake, randomization arm, family history of any cancer and marital status.

In sensitivity analysis, there was little change in the findings after excluding cases who were diagnosed within the first two years of follow-up (all *P* for trend > 0.05). We also performed another sensitivity analysis by dividing the data into two parts (high versus low intake). No significant associations were observed for any types of tomatoes or lycopene (Supplementary Table 1, all *P* > 0.05). There was no statistical evidence for nonlinearity according to the spline curve which is shown in Supplementary Figure 1 (*P* for nonlinearity > 0.05).

## DISCUSSION

In this large PLCO study, we did not observe a significant association between bladder cancer risk and dietary intakes of raw tomatoes, tomato products or lycopene after adjusting for confounders. Similar results were found after excluding cases diagnosed within the first two years of follow-up.

Several case-control studies of dietary tomato/lycopene consumption or serum lycopene (as a marker of consumption of tomatoes and tomato-based products) and the risk of bladder cancer have been published [10, 12, 13, 15] with inconsistent results. Retrospective case-control studies are at risk of selection and recall biases. Two prospective cohort studies by Michaud et al. [11] and Park et al. [16] found that dietary lycopene intake was not related to the bladder cancer incidence in the ATBC cohort study and in the Multiethnic Cohort Study, respectively.

Intake of tomato and/or lycopene has been associated with reduced risk of several cancers, such as hepatocellular carcinoma [17], gastric cancer [18] and prostate cancer [19]. It has been proposed that lycopene, found in high amounts in tomato, may contribute to cancer prevention, which could be the biological mechanisms in the lower development of cancers with higher consumption of tomato, [20]. Owing to its potent antioxidant properties, lycopene can reduce potentially harmful proinflammatory mediators and modulate the downstream cellular signaling [21]. Okajima et al. [22] reported that tomato juice, presumably containing lycopene and other anti-oxidants, inhibited the development of bladder cancer in a rat model.

The strengths of this study include the prospective design, large sample size and a high and complete follow-up rate, which substantially decreased the chance of reverse causality and selection bias. The collected data on smoking status and many other potential confounders made the adjustment as comprehensive as possible. However, several limitations should also be discussed. First, as this was an observational study, causality can only be suggested and residual or unmeasured confounding cannot be fully excluded. Second, the most participants analyzed in this study were non-Hispanic Whites, and as such our findings may not be applicable to other populations (e.g., Asians). Third, it is inevitable that errors commonly exist in nutritional exposures measured by dietary history questionnaire, which may distort the true risk estimates. Finally, participants’ information was collected at baseline only and the exposures could have changed during the long follow-up period.

In summary, analysis of the PLCO study suggested that dietary consumption of tomato or lycopene was not related to the incidence of bladder cancer. Future large prospective studies with detailed information on tomato preparation, molecular subtypes of bladder cancer and genotypes of population could provide more definitive conclusion on the potential effects of tomato or lycopene intake on risk of bladder cancer.

## MATERIALS AND METHODS

### Subjects and study design

PLCO screening trial is a population-based clinical trial aimed to determine whether certain screening tests would reduce death from prostate, lung, colorectal, and ovarian cancer [23]. PLCO consisted of 154,897 eligible participants aged 55 to 74 years and enrolled at 10 screening centers across the United States from 1993 to 2001. The institutional review boards of the National Cancer Institute (NCI) and each of the participating centers approved the PLCO study. All eligible participants provided informed consent in the study.

### Data collection and dietary assessment

The baseline questionnaire (BQ) included participants’ self-reported information on demographics (e.g., age, gender and race), medical history, and other factors. Dietary data were collected using the dietary history questionnaire (DHQ), which included the portion size and frequency of 124 individual food items and supplement use in the past year [24]. The USDA 1994 to 1996 Continuing Survey of Food Intakes by Individuals [25] were used to calibrate DHQ data and calculate the daily intake of raw tomatoes, tomato catsup, tomato salsa, tomato juice and lycopene.

### Subject selection

**Figure 1:**
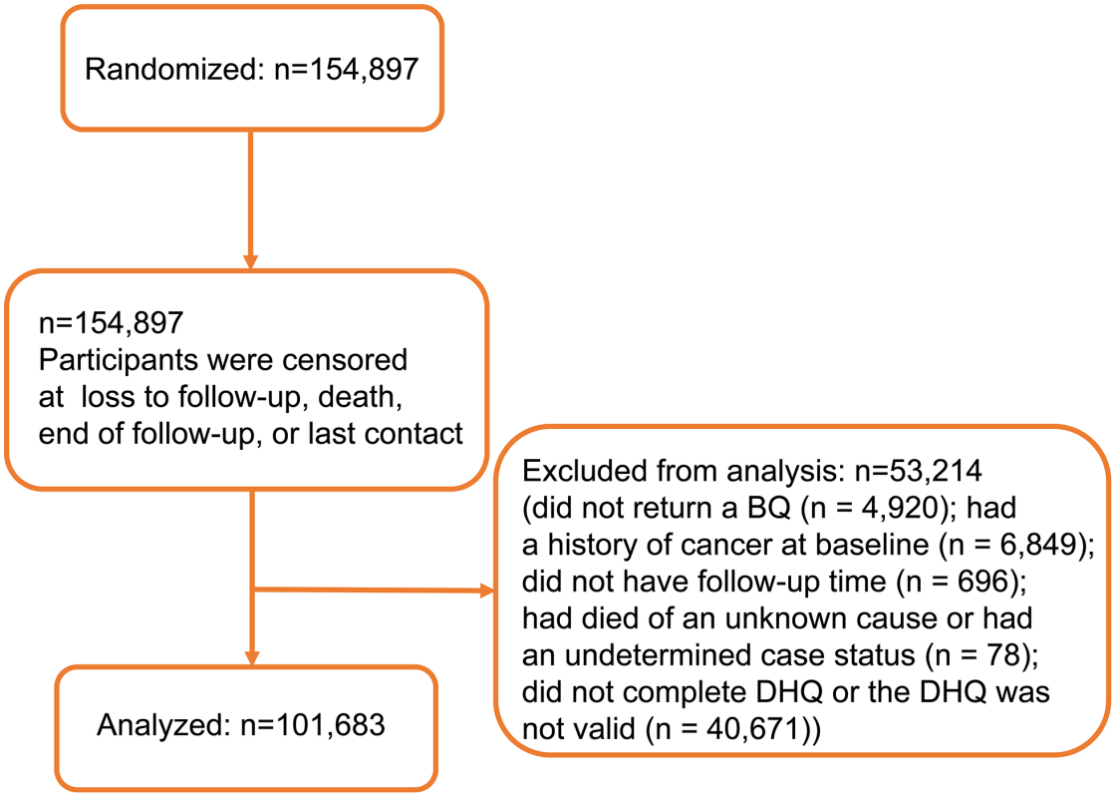
Flow diagram of participant inclusion.

Participants were excluded from this study if they did not return a BQ (n = 4,920); had a history of cancer at baseline (n = 6,849); had no data on follow-up time (n = 696); had died from unknown causes or had an undetermined case status (n = 78); did not complete DHQ or the DHQ was not valid (n = 40,671). Finally, this study comprised 101,683 participants in total. The detailed process of subject selection has been shown in Figure 1.

### Outcome assessment

Study participants were mailed a questionnaire each year to identify cancer cases. Diagnosis of cancer was then ascertained via medical record abstraction. Information on vital status was also supplemented by periodic linkage to the National Death Index. The primary endpoint of interest was the incidence of bladder cancer.

### Statistical analysis

We used Cox proportional hazards regression to estimate HRs and 95% CIs. Participants were censored until cancer diagnosis, occurrence of death, or end of follow-up (December 31, 2009). Models were adjusted for randomization arm (intervention vs. control), age (continuous), sex, race (White, Non-Hispanic vs. Other), BMI (< 25 vs. ≥ 25 kg/m^2^), education (≤ high school vs. ≥ some college), smoking status (never vs. former ≤ 15 years since quit vs. former > 15 years since quit vs. former with years since quit unknown vs. current smoker ≤ 1 pack per day vs. current smoker >1 pack per day vs. current smoker with intensity unknown), drinking habits (never vs. former vs. current), total energy intake (continuous), family history of any cancer (yes vs. no), and marital status (married vs. not married). Interaction was examined using likelihood-ratio tests compared models with and without the interaction term including age, sex, race, education level, drinking habits, smoking status and BMI. The Schoenfeld residuals were used to check the proportional hazards (PH) assumption [26]. Dietary tomato/lycopene consumption was categorized into quintiles before fitting into the models. Restricted cubic spline models [27] were used to examine a potential non-linear association between tomato intake and bladder cancer incidence with three fitted knots (i.e., 10th, 50th and 90th percentiles). All statistical analyses were performed using the software STATA version 15 (Stata Corp, College Station, TX, USA). All tests were two-sided.

## Supplementary Material

Supplementary Figure 1

Supplementary Table 1
