# Supplementary material for: Association of dietary tomato intake with bladder cancer risk in a prospective cohort of 101,683 individuals with 12.5 years of follow-up
Source: Aging (Albany NY). 2021 Jul 9;13(13):17629–37. doi: 10.18632/aging.203252 (PMC8312424; doi:10.18632/aging.203252)
Supplement: Supplementary Figure 1 [file aging-13-203252-s001.pdf]

SUPPLEMENTARY FIGURE

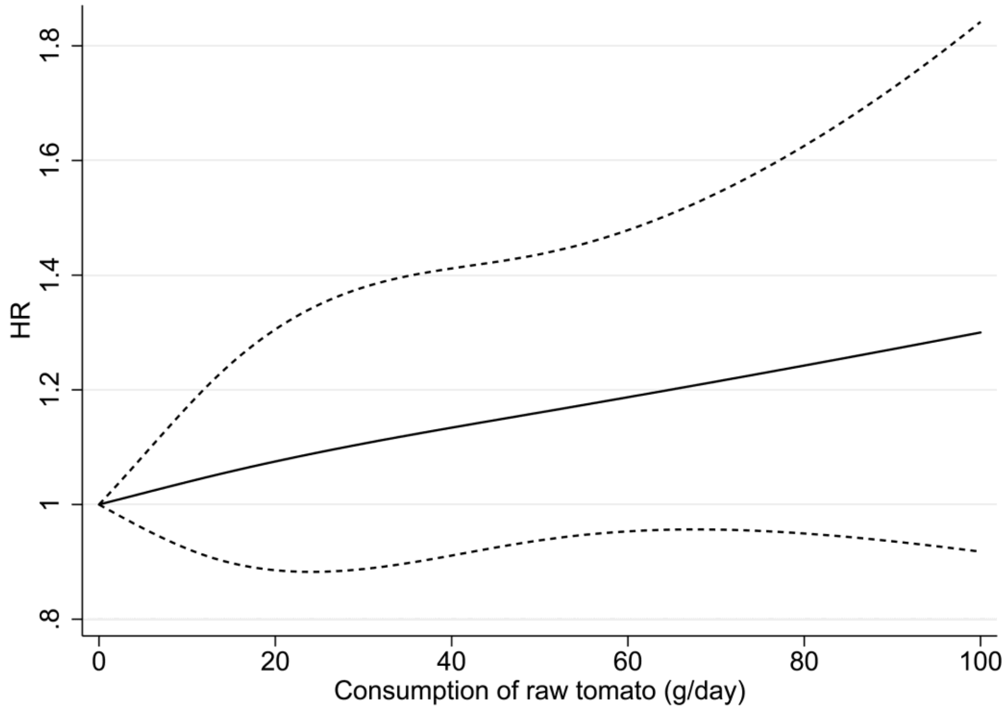

Supplementary Figure 1. Dose-response analysis was performed using restricted cubic spline model for the association between dietary raw tomato intake and bladder cancer risk with three fitted knots (i.e., 10th, 50th, and 90th percentiles) adjusting for age, sex, race, body mass index, education, smoking status, drinking status, marital status, family history of cancer, arm, and total energy intake. Solid lines represent point estimates and dashed lines represent 95% confidence intervals.
