# Supplementary material for: Association of dietary tomato intake with bladder cancer risk in a prospective cohort of 101,683 individuals with 12.5 years of follow-up
Source: Aging (Albany NY). 2021 Jul 9;13(13):17629–37. doi: 10.18632/aging.203252 (PMC8312424; doi:10.18632/aging.203252)
Supplement: Supplementary Table 1 [file aging-13-203252-s002.pdf]

SUPPLEMENTARY TABLE

Supplementary Table 1. Association between tomato/lycopene intake and bladder cancer risk (High vs. Low).

Variables	Raw tomato	Tomato catsup	Tomato salsa	Tomato juice	Lycopene
Adjusted HR (95% CI)*	1.03 (0.89-1.19)	1.02 (0.88-1.18)	0.98 (0.85-1.14)	0.95 (0.82-1.10)	0.99 (0.85-1.16)
p-value	0.653	0.808	0.812	0.503	0.941

HR, hazard ratio; CI, confidence interval.

*Adjusted for age, sex, race, body mass index, education, smoking status, drinking status, total energy intake, randomization arm, family history of any cancer and marital status.
